# Immune Dysregulation in Patients Persistently Infected with Human Papillomaviruses 6 and 11

**DOI:** 10.3390/jcm4030375

**Published:** 2015-03-03

**Authors:** Alexandra V. Lucs, James A. DeVoti, Lynda Hatam, Ali Afzal, Allan L. Abramson, Bettie M. Steinberg, Vincent R. Bonagura

**Affiliations:** 1 Feinstein Institute for Medical Research, Manhasset, NY 11030, USA; E-Mails: alucs@nshs.edu (A.V.L.); JDevoti@nshs.edu (J.A.D.); lhatam@nshs.edu (L.H.); Raliafzal@nshs.edu (A.A.); aabramso@nshs.edu (A.L.A.); bsteinbe@nshs.edu (B.M.S.); 2 Division of Allergy and Immunology, Department of Pediatrics, Hofstra North Shore-LIJ School of Medicine, Great Neck, NY 11549, USA; 3 Elmezzi Graduate School of Molecular Medicine, Manhasset, NY 11030, USA; 4 Department of Otolaryngology, Hofstra North Shore-LIJ School of Medicine, New Hyde Park, NY 11549, USA; 5 Department of Molecular Medicine, Hofstra North Shore-LIJ School of Medicine, Manhasset, NY 11549, USA

**Keywords:** HPV, recurrent respiratory papillomatosis, anogenital warts, innate immunity, Langerhans cells, T cells, natural killer cells

## Abstract

Human Papillomaviruses (HPVs) 6 and 11 are part of a large family of small DNA viruses, some of which are commensal. Although much of the population can contain or clear infection with these viruses, there is a subset of individuals who develop persistent infection that can cause significant morbidity and on occasion mortality. Depending on the site of infection, patients chronically infected with these viruses develop either recurrent, and on occasion, severe genital warts or recurrent respiratory papillomas that can obstruct the upper airway. The HPV-induced diseases described are likely the result of a complex and localized immune suppressive milieu that is characteristic of patients with persistent HPV infection. We review data that documents impaired Langerhans cell responses and maturation, describes the polarized adaptive T-cell immune responses made to these viruses, and the expression of class select II MHC and KIR genes that associate with severe HPV6 and 11 induced disease. Finally, we review evidence that documents the polarization of functional T_H_2 and T-regulatory T-cells in tissues persistently infected with HPV6 and 11, and we review evidence that there is suppression of natural killer cell function. Together, these altered innate and adaptive immune responses contribute to the cellular and humoral microenvironment that supports HPV 6 and 11-induced disease.

## 1. Introduction

Human papillomaviruses (HPVs) comprise a large family of viruses which are considered to be part of the normal flora of the human epithelium. Periodically these commensal viruses cause benign growths, resulting in warts (cutaneous) or papillomas (mucosal). However, most immune competent people are capable of clearing or containing, periodic flares of active HPV infection, and the virus remains in a latent state for most or all of the life of the host. The most infamous of these viruses are HPV types 16 and 18, the prototypical “high-risk virus” that cause over 70% of cervical cancers and are known for their roles in other anogenital and, more recently, oropharyngeal cancers. HPVs 6 and 11, known as “low-risk viruses” because of the rarity with which they induce cancers, also impose a heavy burden to society. They are responsible for most cases of anogenital warts (genital warts), which have an annual worldwide incidence of 195 per 100,000 [1]. Despite the lack of serious medical complications, the negative psychosocial impact of genital warts causes most people to seek treatment, creating an economic burden for the economy. HPVs 6 and 11 can also cause a rare, but on occasion, a much more severe disease, known as recurrent respiratory papillomatosis (RRP). As in genital warts, the HPVs cause papillomas to form, but in this case in the upper airway, primarily the larynx. The narrowed airway becomes obstructed by papilloma tissue and these patients, if left untreated, can asphyxiate. Moreover, patients with severe RRP may require greater than 150 surgical procedures to maintain a patent airway, with an associated annual cost of $100 million US dollars [2].

A subset of patients with genital warts and respiratory papillomas exhibit multiple recurrences of large papillomas, indicating that there is an inability of the immune system of these patients to clear or contain this infection. However, analysis of the lymphocyte phenotypes found in peripheral blood mononuclear cells (PBMC) of either set of patients reveals no significant difference from the lymphocyte populations found in normal controls [3,4], indicating that any immune dysfunction is likely to be contained to the site of HPV infection. Indeed, a long list of papers examining immune cells at these sites have found defects in Langerhans cells, enrichment of T regulatory (Treg) cells, skewing of the T helper cells to the T_H_2 phenotype, and natural killer (NK) cell dysfunction. The obvious alterations in the localized immune environment, combined with the normal immune profile in the PBMC, indicate that there is a localized immune suppression induced by HPVs 6 and 11 in these patients. Although mechanisms of immune evasion of HPV16 have been described and thoroughly reviewed [5,6,7], there exists, to date, no comprehensive review of the immune suppression in HPV 6 and 11-induced papillomas. Here we will combine data from genital warts and RRP to assemble the known phenotypes and mechanisms of immune dysfunction found in HPV 6 and 11 persistent infections.

**Figure 1:**
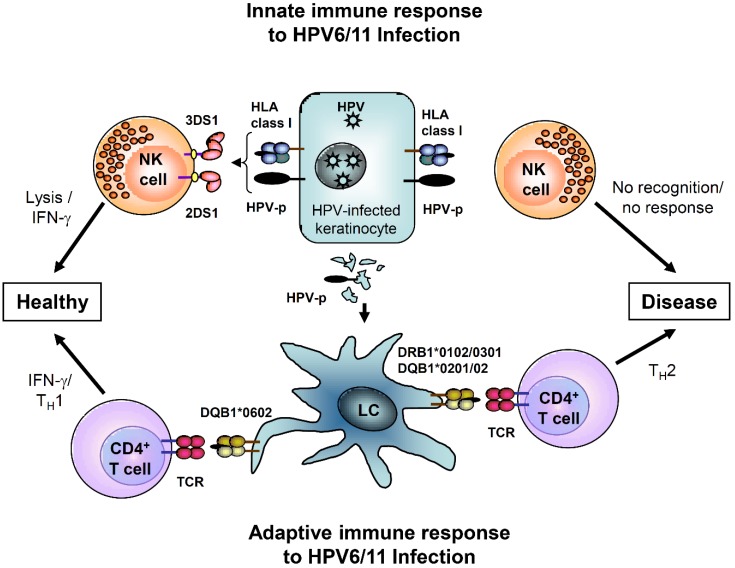
Immune Responses Support Persistent HPV 6/11 infection that Causes Human Papillomaviruses (HPV)-induced Disease. Both the innate and adaptive immune responses to HPV infection are polarized away from effective T_H_1-like responses in genital warts and respiratory papillomas, leading to persistent HPV-induced disease. The innate immune response to HPV infection is normally mediated by Natural Killer (NK) cell recognition of activating and inhibited killer cell immunogloublin-like receptor (*KIR*) gene products that recognize reduced human leukocyte antigen (HLA) class I molecules on HPV-infected keratinocytes. Lack of recognition, due to activating haplotype biasing in recurrent respiratory papillomatosis (RRP), results in an infective innate immune response. The adaptive immune response is also polarized by the failure of Langerhans cells to mature and then present peptides to T-cells, which results in altered/impaired Langerhans cell (LC) signaling that results in a T_H_2 T-cell bias, which when combined with the lack of NK activity, results in persistent HPV-induced disease. Figure adapted from [14].

We have extensively studied the adaptive cellular responses and major histocompatibility (MHC) gene expression of patients with RRP who have persistent HPV 6 and 11 infection [8,9,10]. These responses are associated with select class II MHC expression that clearly polarizes T-cell responses towards T_H_2-like regulatory T-cell (Treg) function [11,12,13]. These responses generate T_H_2-like cytokine and chemokine expression that is characteristic of patients with severe RRP [10]. Moreover, we have reported that patients with RRP have a restricted natural killer cell immunoglobulin-like receptor (*KIR*) gene haplotypes that associates with increased NK cell numbers with reduced killing capacity [14]. These skewed adaptive and innate responses are demonstrated in patients with RRP, and may be similar to HPV 6/11 induced disease at other anatomical sites. Figure 1 is a schematic of the alteration in adaptive and innate responses that would lead to HPV 6/11-induced disease at mucosal and non-mucosal sites of infection with these viruses.

**Figure 2:**
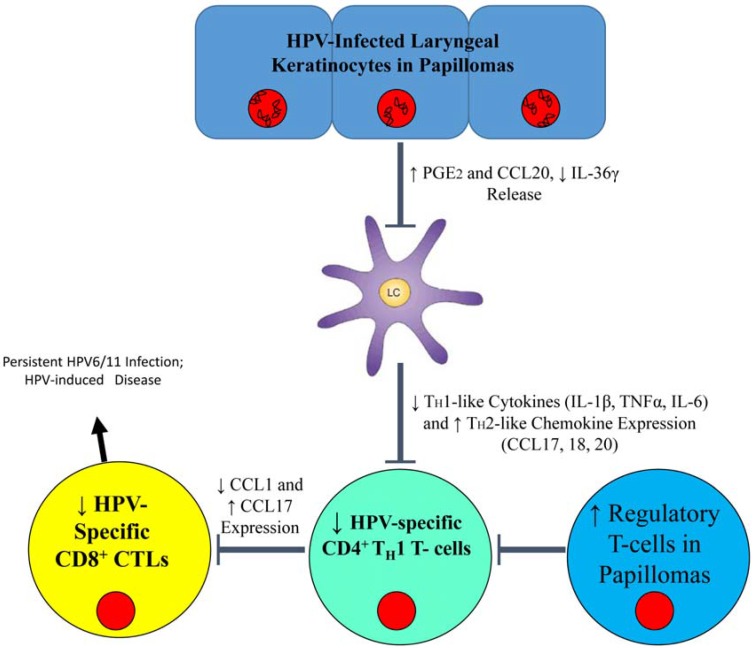
Innate Signaling of HPV 6/11-infected Keratinocytes Modulates the Immune Response in RRP. Keratinocytes harboring persistent HPV-infection have increased levels of PGE_2_ and CCL20, while levels of the pro-inflammatory cytokine IL-36 γ are decreased. The alterations in this cytokine milieu inhibit the normal activation of Langerhans cells, which in turn reduces the number of HPV-specific T_H_1 cells in the lesion and supports regulatory T-cell differentiation from T_H_0 T-cells. In addition, the localized polarization of T cells toward a T_H_2 phenotype reduces the T_H_1 population in the lesion. Lack of HPV-specific T_H_1 cells, results in low levels of HPV-specific CD8+ cells that supports a micromilieu of immune suppressive cells, chemokine, and cytokines that supports persistent HPV infection.

More recently, we have begun the study of the role played by innate immune signaling in the polarization of adaptive immune responses that we have characterized in patients with RRP. Figure 2 summarizes our present understanding of the defect in immune responses made in the epithelium of HPV 6/11 infected individuals with RRP that polarizes the adaptive immune responses, which we hypothesize may be similar in individuals infected with these HPVs at other anatomical sites.

## 2. Langerhans Cells

Langerhans cells (LCs) are the principle subset of dendritic cells found in the epithelial tissue. They are migratory cells that specialize in uptake, transport, processing, and presentation of antigens to T-cells [15,16,17,18]. Immature LCs are essential in generating regulatory T-cells that induce tolerance *in vitro* and *in vivo* [19,20]. LCs mature during antigen encounter, and migrate to lymph nodes [21] where they present peptides to T-cells. LCs are known targets of multiple viral infections. HIV directly infects LCs, while HSV infection of keratinocytes results in a downregulation of LC costimulatory molecules and resistance to maturation stimuli such as LPS [22]. HPVs have been thought to escape recognition by the Langerhans cells, as they fail to induce costimulatory molecules and maturation markers [23]. And yet, excepting the β genus, most HPVs induce a clearance of Langerhans cells (LCs) from the site of infection, preventing T-cell activation. The noticeable exceptions to this pattern are HPVs 6 and 11 [24,25]. Although most authors agree that LC numbers are reduced in lesions caused by 6 and 11, the reduction is nowhere near as dramatic as the almost complete disappearance of LCs observed in HPV-induced cervical disease [25,26]. However, the role of the LCs in control of HPV 6 and 11 infections is still unclear. Multiple attempts to correlate LC presence with regression of genital warts demonstrate no difference in Langerhans cell number in regressing and non-regressing warts [27,28], with one exception that showed lower numbers of LCs in non-regressing [29]. Closer analysis, however, reveals clear indications that the LCs present in HPV 6 and 11-induced lesions have altered distribution and morphology, indicating impaired function. In contrast to LCs in normal tissue, which are evenly distributed in the tissue and have characteristic dendritic processes, Langerhans cells in both genital warts and laryngeal papillomas are found in clumps toward the basal layer of the epidermis and dendritic processes are shortened or absent [30,31]. Moreover, we have shown that LCs in papillomas lack the maturation marker CD83 [32]. Therefore, it is clear that the LCs present in HPV 6 and 11-induced lesions are not classically activated.

There is some debate about whether the LCs present in HPV 6 and 11-induced lesions are simply nonfunctional or anergic, or if they have an active role in the pathogenesis of the disease. Clinical trials treating genital warts with the anti-viral agent imiquimod showed decreases in CD1a+ cells [33], indicating that LCs left the tissue and did not return in response to this treatment. Murine studies confirmed that imiquimod induces migration of LCs to the draining lymph nodes, but interestingly, they showed that neither the costimulatory factor CD80 or CD86 was upregulated [34], bringing into question the functionality of these cells.

The cytokines made by keratinocytes [35,36,37,38], with contributions from LCs themselves, are critical in influencing LC function. The balance between pro-inflammatory cytokines and the anti-inflammatory cytokine IL-10 likely regulate LC migration [38]. Additionally, TGF-β expression is critical for immature LC maintenance and maturation [39]. While LCs can induce T_H_1 responses, they can be converted to support T_H_2 responses when IL-10 is present [21,40,41,42,43,44,45,46,47]. IL-10 and TGF-β inhibit the differentiation of LCs and DCs, and IL-10 blocks T_H_1 responses have been shown to induce tolerance to tumors [48], while preserving LC and DC induction of T_H_2 responses to antigens [20]. Thus, LCs can function as either pro- or anti-inflammatory mediators of adaptive immunity [49,50,51,52,53,54].

Although the small biopsy sample sizes in RRP have to date made functional studies of LCs from these tissues difficult, monocyte-derived immature Langerhans cells from RRP patients show an attenuated response to the proinflammatory cytokine IL-36γ expressed by keratinocytes [32], suggesting that these cells may be less functional. The reduced LC response may be a general response to HPV infection, as reported in a recent study of LCs from cervical cancer, which showed a lack of cytokine expression when LCs were stimulated with Toll 7 or 8 ligands, suggesting that LCs can become anergic during active HPV infection [55]. However, in 2008, Cao *et al.* proposed that the LCs in genital warts are not simply inactive, but instead are the sources of CCL17 and CCL22, cytokines shown to recruit Tregs to local tissues [3,56]. Both by co-staining experiments and by qPCR they showed that LCs produce these cytokines, and furthermore, they demonstrated that antibody-induced blockade of either CCL17 or CCL22 inhibits migration of Tregs towards wart tissue in culture. The proposed mechanism of Treg recruitment is consistent with the data found in RRP which shows that CCL17 and CCL22 are produced by papilloma tissues and disseminated widely enough to be found in plasma [10,12]. Moreover, remission of RRP patients in a clinical trial of celecoxib occurs in concert with decreased plasma levels of these chemokines [10]. If it can be confirmed that LCs are the source of CCL17 and CCL22 in HPV 6 and 11-induced papillomas, it would suggest that LCs play an active role in creating and maintaining local immune suppression by recruiting T regulatory cells into HPV 6 and 11 infected epithelial tissues.

## 3. T Cells

Tregs, increased in many different cancer tissues, are known to be suppressors of the immune response and they are both present and functional in genital warts and respiratory papillomas. FOXP3^+^ Tregs isolated from either site suppressed PBMC proliferation in micro co-culture assays [3,12]. In genital warts, size of the wart correlated with the relative abundance of Tregs (<1% of all T cells in small warts and >6% in large warts [3]. Direct assessment of the effects of Treg depletion was attempted in a small clinical trial in patients with condylomata acuminata using cyclophosphamide, a common chemotherapeutic drug known to block proliferation of dividing cells by interfering with DNA replication, to treat patients for one week after warts were removed by laser therapy. The obvious caveat to this study is the lack of specificity of this drug. The authors suggest that low doses of this drug resulted in selective depletion of the Treg population, but only showed that the NK population is not depleted in response at these low doses. Given these caveats, the clinical trial did show that low-dose cyclophosphamide treatment depletes Tregs and increases T cell proliferation and cytolysis. The net effect of the treatment was a decrease in detectable HPV DNA and in wart recurrence. Thirty weeks after treatment only 17% of patients had recurrence of disease compared with 81% in the placebo group [57]. Further experiments need to be performed to show that the non-recurrence observed in this trial is truly due to the depletion of Tregs, but it does lend support to the predominate theory held in the field that the high proportion of Tregs found in HPV 6 and 11-induced lesions actively suppresses the HPV-specific immune response and allows for the development and recurrence of disease.

It should be noted that Tregs comprise only a subset of the CD4+ T cells in HPV 6 and 11 induced lesions, being approximately 6% in genital warts and 25% in respiratory papillomas [12,57]. However, we have shown that in papillomas half of the CD4+ T cell population express very low levels of CD127, indicating that these cells, although present, are exhausted [12]. Although direct analysis of T_H_1 *vs.* T_H_2 cells within the CD4+ T cell subpopulation in the tissues has not been completely characterized in sites infected with HPV 6/11, IL-10 and TGF-β, which are markers of T_H_2-like cells, have been shown to be highly expressed in genital warts and laryngeal papillomas [3,8,13,58]. There is also evidence of T_H_2-like polarization of the adaptive immune response to HPV 6/11 in laryngeal papillomas and in the peripheral blood. Genital wart patients have been shown to actually have an increased ratio of T_H_2/T_H_1 cells in PBMC, while RRP patients have an increase in serum levels of T_H_2 chemokines [10,59].

As a final note on T cells, CD8+ T cells are present in both genital warts and respiratory papillomas [8,27]. These cells are likely to be immature cytotoxic T-cells, suggesting that the dominant T_H_2/Treg microenvironment in respiratory papillomas has an impact on CD8+ T-cell maturation that further prevents HPV clearance or suppression of HPV6/11-infected keratinocytes [8,32].

## 4. Natural Killer Cells

Class I MHC expression is markedly reduced or absent in laryngeal papillomas [4], and natural killer (NK) cells present in papilloma tissues should remove keratinocytes that lack class I MHC expression. However, the NK cells could be inhibited by the high proportion of Tregs in the lesions since Tregs are known to regulate NK activity [60]. Moreover, studies suggest that suppression of NK cell function is not restricted to the HPV-induced lesions since NKs derived from PBMC of patients with genital warts or RRP also exhibit suppressed cytolytic activity [14,61]. The mechanism for this suppressed activity is thought to be decreased production of IL-2 and IFNγ.

Interestingly, the frequency of killer cell immunogloublin-like receptor (*KIR*) gene haplotypes, which control natural killer cell cytotoxicity to viral infection, show differences between controls and RRP patients. In addition, it has been shown that HLA class II alleles DRB1*0102, DRB1*0301, DQB1*0201 and DQB1*0202 confer susceptibility to RRP [4] and that patients expressing these alleles are less likely to have the activating the KIR genes 2DS1 and 3DS1 in their NK haplotype, a correlation that grows increasingly significant with the severity of the disease [14]. These studies suggest that there is likely to be a genetic predisposition to develop persistent HPV6 and 11 infection and more so, severe disease.

## 5. Discussion

For decades laboratories that study genital warts and RRP have been characterizing a series of localized immune deficiencies in HPV 6- and 11-induced lesions. We have discovered that LCs are present, but have an altered distribution and morphology, are immature in respiratory papillomas, and that T cells in these lesions appear to have a T_H_2/Treg bias. Furthermore, the relatively large proportion of Tregs in these lesions suppresses NK cells so that they fail to compensate for defective effector T-cell function. As we learn more about each of the functions of these immunocytes in patients with persistent HPV 6 and 11 infections, a new paradigm is developing to explain the deviation in immune regulation that supports this process. Langerhans cells express chemokines that recruit T_H_0 T-cells that can differentiate into inducible Tregs as well as recruiting natural Tregs to papillomas. Both types of Tregs can polarize naïve T_H_0 T-cells to become T_H_2 cells that can inhibit NK cell function, thus creating a localized cellular and cytokine/chemokine micromilieu that can block the T_H_1-like effector T-cell responses that should clear or at least control HPV 6/11 activation (Figure 1).

What remains unclear is the role for HPV 6 and 11 in this process, other than in exploiting this immunosuppressed microenvironment. Studies of HPV 16 indicate that early protein E6 and E7 are responsible for downregulating IFN-γ signaling and regulate Cox-2 transcription [62], however it is well known that HPV16 E6 and E7 proteins have required functions that are unique to HPV 16 successful replication. The dearth of HPV 6 and 11-specific information regarding the role of these early proteins in skewing immune function is due to the lack of an animal model in which to study HPVs 6 and 11. The vast majority of what we have learned about the localized immune suppression was discovered by analyzing cells from tissue biopsies, either immediately or after brief passage in cell culture, and the use of PBMC stimulated by HPV 6/11-infected tissues or purified HPV early proteins [8,58]. Although this has afforded us great insights into the function of the immune cells in the blood or tissue responding to HPV 6 and 11, these studies are not conducive to manipulation of viral proteins. However we do know that HPV E7 blocks MHC class I expression [63,64] and E6 has been shown to promote T_H_2-like T-cell polarization of peripheral blood mononuclear cells [8].

It is clear that HPVs 6 and 11 alone are not solely responsible for this local immune suppression, and that a defect(s) in host response is essential in development of persistent HPV infection. Selective enrichment of HLA class II MHC genotypes (DRB1*0102, DRB1*0301, DQB1*0201 and DQB1*0202) and restricted KIR allele haplotypes (2DS1 and 3DS1) in the RRP population indicate that there is indeed a genetic predisposition of these patients to develop RRP and severe disease [4,14]. It is also possible that the decreased cytolytic function of NK cells and absence of mature HPV-specific CD8+ T-cells in PBMC and in HPV-induced lesions may be important host susceptibility factors independent of the cytokines secreted by Tregs. More experiments are needed to define the role of each of these observations.

Although we have focused in this review on the many similarities of immunosuppression in patients with genital warts and RRP, it is notable that some differences do exist. Tregs are found in both tissues, but in significantly different proportions, as stated above: 6% of CD4+ T cells in genital warts, and as many as 25% of CD4+ T cells in laryngeal papillomas. In genital warts the proportion of Tregs increases with the size of the wart. It may be that only the most severe of the laryngeal papillomas were large enough to provide tissues for analysis, and thus we selected papillomas with the highest possible proportion of Tregs. However, this is unlikely since not all of the large papillomas we assayed contained high Treg numbers. In addition, it has been reported that expression of TLRs 2, 3, 4, 7, 8 and 9 is upregulated in genital warts [65] while our data showed no indication of changes in TLR expression in laryngeal papillomas [66]. This may be due to differences in TLR expression in keratinocytes at different anatomical locations.

## 6. Conclusions

Despite the minor variations in immune suppression observed in RRP and genital warts, it is clear in both these diseases that persistent HPV 6 and 11 infections are most likely the result of impaired innate immune responses made by resident Langerhans cells and natural killer cells. This permissive innate response would lead to a polarization of the adaptive T-cell immune response towards an unapposed T_H_2/Treg bias in the T cell population present in both patients with genital warts and RRP (Figure 2). The major questions that remain are what the direct effects of individual HPV early proteins on this immune dysfunction, and what novel medical strategy could be used to effectively modulate the defective host responsiveness. Ultimately, further understanding is needed of why only a select few of the individuals fail to contain or eliminate acute HPV6/11 infection in the larynx or perineum and are subject to persistence or recurrent activation. The long term goal of these studies is to better understand the innate and adaptive immune dysregulation induced by HPV6/11 in individuals who show persistent infection and thereby improve the current treatment strategy for these diseases.
